# Iron-rich Smectite Formation in Subseafloor Basaltic Lava in Aged Oceanic Crust

**DOI:** 10.1038/s41598-019-47887-x

**Published:** 2019-08-05

**Authors:** Seiya Yamashita, Hiroki Mukai, Naotaka Tomioka, Hiroyuki Kagi, Yohey Suzuki

**Affiliations:** 1 Department of Earth and Planetary Science, The University of Tokyo, 7-3-1 Hongo, Bunkyo-ku, Tokyo Japan; 2 Kochi Institute for Core Sample Research, Japan Agency for Marine-Earth Science and Technology (JAMSTEC), 200 Monobe Otsu, Nankoku, Kochi 783-8502 Japan; 3 Geochemixal Research Center, The University of Tokyo, 7-3-1 Hongo, Bunkyo-ku, Tokyo 113-0033 Japan

**Keywords:** Mineralogy, Physical oceanography

## Abstract

Basalt weathering in oceanic crust controls long-term elemental cycling on Earth. It is unknown whether basalt weathering tends to continue in unsedimented oceanic crust with formation ages of >10–20 million years (Ma), when fluid circulation is restricted by the formation of secondary minerals in fractures/veins. We investigated basalt weathering in 13.5-, 33.5- and 104-Ma oceanic crust below the South Pacific Gyre by combining bulk and *in-situ* clay mineral characterisations. Here we show the formation of iron-rich smectite at the rims of fractures/veins in 33.5-Ma and 104-Ma core samples from depths as great as 121 metres below the seafloor. In contrast, iron-rich smectite formation was not observed in three 13.5-Ma core samples, which suggests that iron-rich smectite formation may be affected by the dilution of aqueous silica supplied from basalt dissolution by actively circulating fluid. As iron-rich smectite from the 33.5-Ma and 104-Ma core samples was more enriched in Mg and K than that typically found at hydrothermal mounds, iron-rich smectite formation appears to result from basalt weathering rather than hydrothermal alteration. Our results suggest that unsedimented basaltic basement is permeable and reactive to host microbial life in aged oceanic crust on Earth and possibly in the deep subsurface on Mars.

## Introduction

Rock**–**water interactions in oceanic crust, an uppermost layer of a tectonic plate, are known to influence the chemical compositions of the atmosphere, hydrosphere and lithosphere^1^. For the biosphere on the surface, it is suspected that cycles of major mass extinctions over several hundred million years are linked to the weathering of oceanic crust, where carbon dioxide has been stored at a massive scale in rock fractures/veins as calcium carbonate minerals and later been released into the atmosphere from volcanoes at subduction zones^2^. The planetary importance of rock**–**water interactions is not limited to the Earth’s crust but applies to other planetary bodies such as Mars^3,4^.

The upper oceanic crust is mainly composed of basaltic lava erupted and solidified at mid-ocean ridges, where the buoyance of heated fluid drives vigorous circulation of oxygenated seawater. As a result of thermally driven fluid circulation, basaltic lava is hydrothermally altered to form secondary minerals such as a mica mineral called celadonite [K(Mg, Fe^2+^)(Fe^3+^, Al)(Si_4_O_10_)(OH)_2_] and iron oxyhydroxides^5^. On the young ridge flank, the circulation of oxygenated seawater is restricted, because thermally driven fluid circulation is weakened by rapid crustal cooling^6^. At this stage, the crustal fluid is enriched with reducing compounds such as Fe(II) and HS^−^, to form pyrite [FeS_2_] and a smectite mineral called saponite [Ca_0.17_Mg_3_(Si, Al)_4_O_10_(OH)_2_·n(H_2_O)]_5_. On the older ridge flank, cold, oxygenated seawater is circulated in the upper oceanic crust with a deficit of the reduced compounds^7^. Further infilling of fractures/veins with calcium carbonate and zeolite substantially reduces porosity and permeability^5^, which appears to be synchronous with a sharp decline in oxidative alteration in cold oceanic crust aged 10–20 Ma^8^.

In addition to the effect of secondary mineralisation on the permeability and chemical reactivity of the upper oceanic crust, build-up of low-permeability sediment isolates buried basaltic lava and thus separates it from the overlying seawater^9,10^. The basaltic portion of the oceanic crust beneath sediment cover is referred to as basaltic basement. Although basaltic basement covered with a thin layer of sediment is spatially vast^11^, it is unknown whether unsedimented basaltic basement is chemically reactive with seawater and influences elemental cycling. During the Integrated Ocean Drilling Project (IODP) Expedition 329 using the drilling vessel *JOIDES Resolution*, we obtained core samples from basaltic basement below the South Pacific Gyre (SPG), where extremely low supplies of planktonic debris and aeolian dust result in basaltic basement thinly covered with sediments^12^. The SPG environment promotes extremely low activity of aerobic microbial communities in the overlying sediments, which enables O_2_ to penetrate the ocean floor down to the basaltic basement^13^. Here, we characterised unsedimented basaltic basement cores collected at Sites U1365, U1367, and U1368 with crustal ages of 104 Ma, 33.5 Ma, and 13.5 Ma, respectively^14–16^ (Supplementary Fig. S1, Supplementary Table S1).

## Results

**Figure 1:**
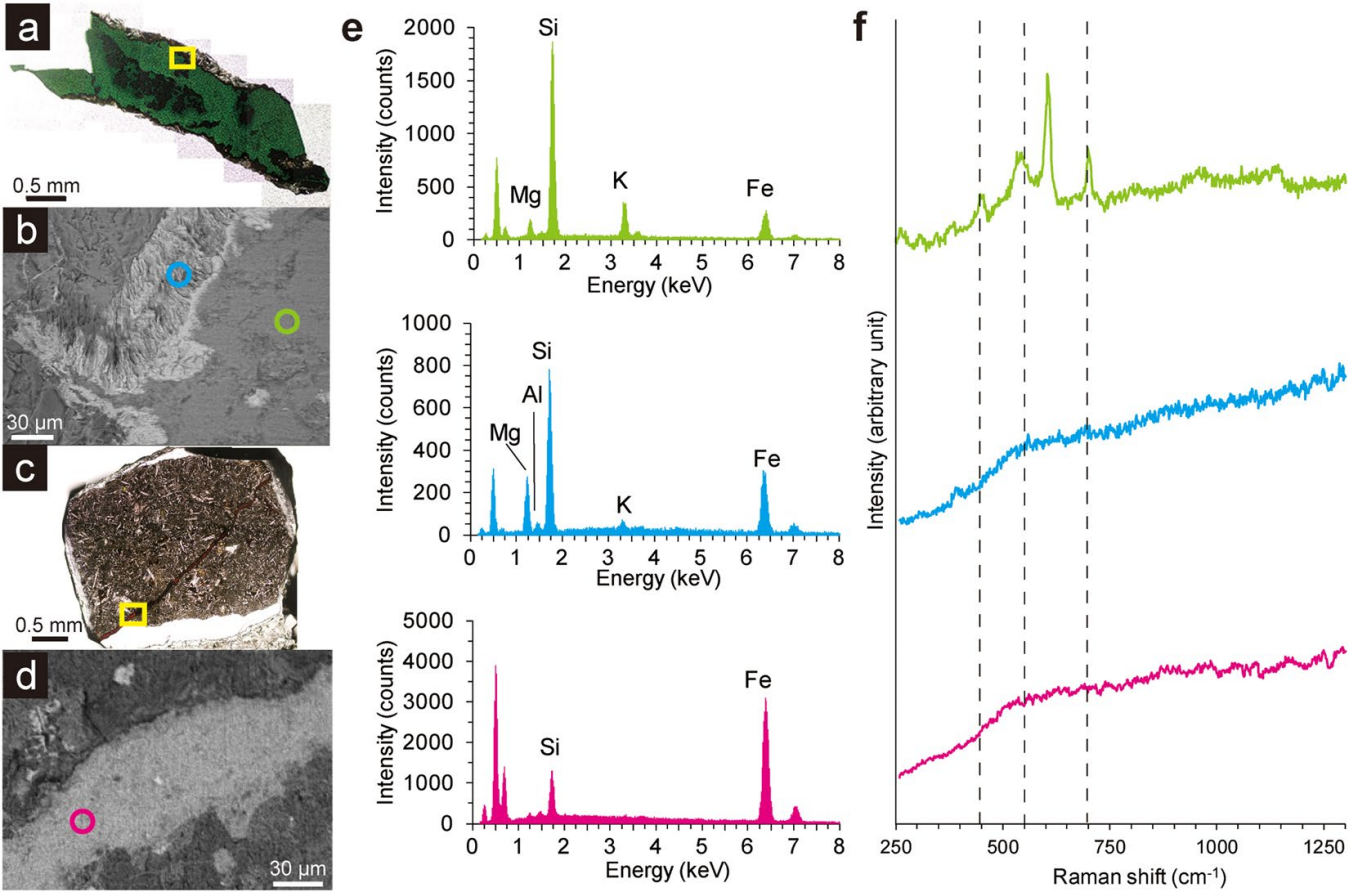
Spectroscopic characteristics of fractures/veins in basaltic basement at Site U1365 (104 Ma). Optical microscopy images and back-scattering electron images (BEI) from U1365E-8R4 (**a**,**b**) and U1365E-12R2 (**c**,**d**). Highlighted areas with yellow rectangles in optical microscopy images correspond to BEI images. (**e**) SEM-EDS spectra with colours obtained from circles with the same colours in BEI. (**f**) Raman spectra obtained from the circles with the same colours in the corresponding BEI. Peak positions of three major peaks in a Raman spectrum of a celadonite standard from RRUFF are shown with dotted lines.

**Figure 2:**
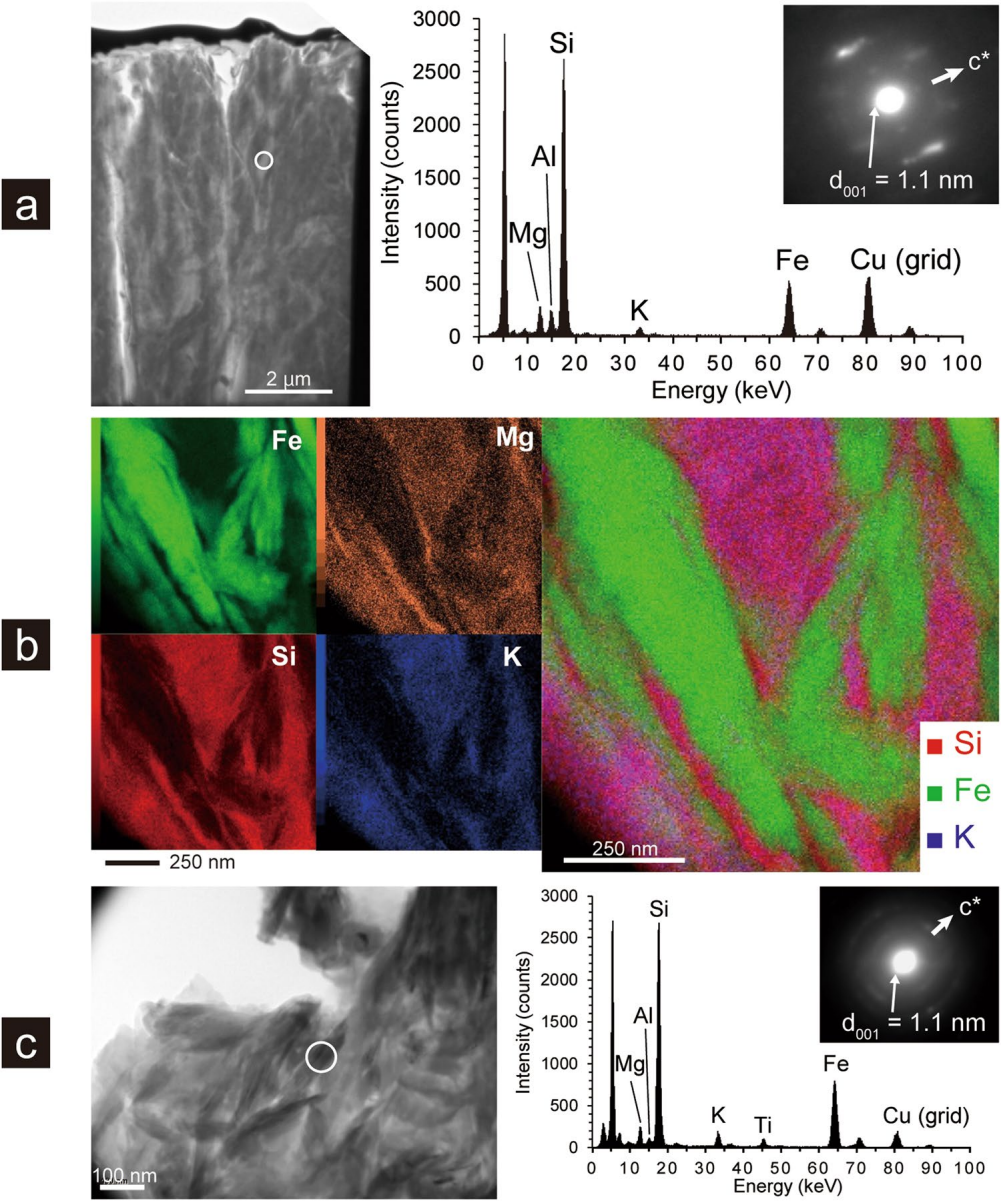
Mineralogical characteristics of Fe-rich phyllosilicate minerals in U1365E-8R4. (**a**) Bright-field TEM micrograph of fibrous material located between a celadonite-filled fracture and the groundmass with a circle, from which an EDS spectrum and an SAED pattern were obtained. (**b**) Elemental mapping images of Fe, Si, Mg, and K by scanning transmission electron microscopy and an RGB synthesis image of Si, Fe, and K. (**c**) Bright-field TEM micrograph of a suspended fraction of a powdered sample with a white circle, from which an EDS spectrum and an SAED pattern were obtained.

We investigated thin sections of basaltic core samples (sample codes: U1365E-8R4 and U1365E-12R2) at depths of 109.6 m and 121.8 m below the seafloor (mbsf), respectively. U1365E-8R4 and U1365E-12R2 were associated with fractures/veins mainly filled with celadonite and iron oxyhydroxides, respectively (Supplementary Table S2). These samples were the oldest and deepest samples obtained in this study for characterising mineral assemblages within fractures/veins. Observations with optical microscopy, scanning electron microscopy (SEM) equipped with energy dispersive X-ray spectroscopy (EDS), and μ-Raman spectroscopy revealed that on-board identification of celadonite and iron oxyhydroxides as fracture-infilling minerals of U1365E-8R4 and U1365E-12R2 was correct (Fig. 1, Supplementary Table S2). A layer with fibrous material mainly composed of Si, Fe, and Mg without a diagnostic Raman spectrum was found between a celadonite-filled fracture and basaltic groundmass in U1365E-8R4 (Fig. 1f). For U1365E-12R2, μ-Raman spectroscopy revealed that iron oxyhydroxides were amorphous or poorly crystalline (Fig. 1f). To identify the fibrous material in U1365E-8R4, 100-nm-thick ultra-thin sections were prepared using focused ion beam fabrication technology and characterised by transmission electron microscopy (TEM) equipped with EDS (Fig. 2a). Selected area electron diffraction patterns from the fibrous material were characteristic of clay minerals with a basal spacing (*d*_001_) of ~1.1 nm. By using scanning TEM, X-ray elemental mapping images were obtained to clarify compositionally homogenous clay packets (Fig. 2b). We performed the same nanoscale mineral characterisations at a vein rim associated with iron oxyhydroxides in U1365E-12R2 (Supplementary Fig. S2). Although the fibrous layer was not recognised by SEM-EDS analysis, the presence of fibrous material compositionally and structurally similar to that observed in U1365E-8R4 was revealed at the rim of the iron oxyhydroxide vein.

**Figure 3:**
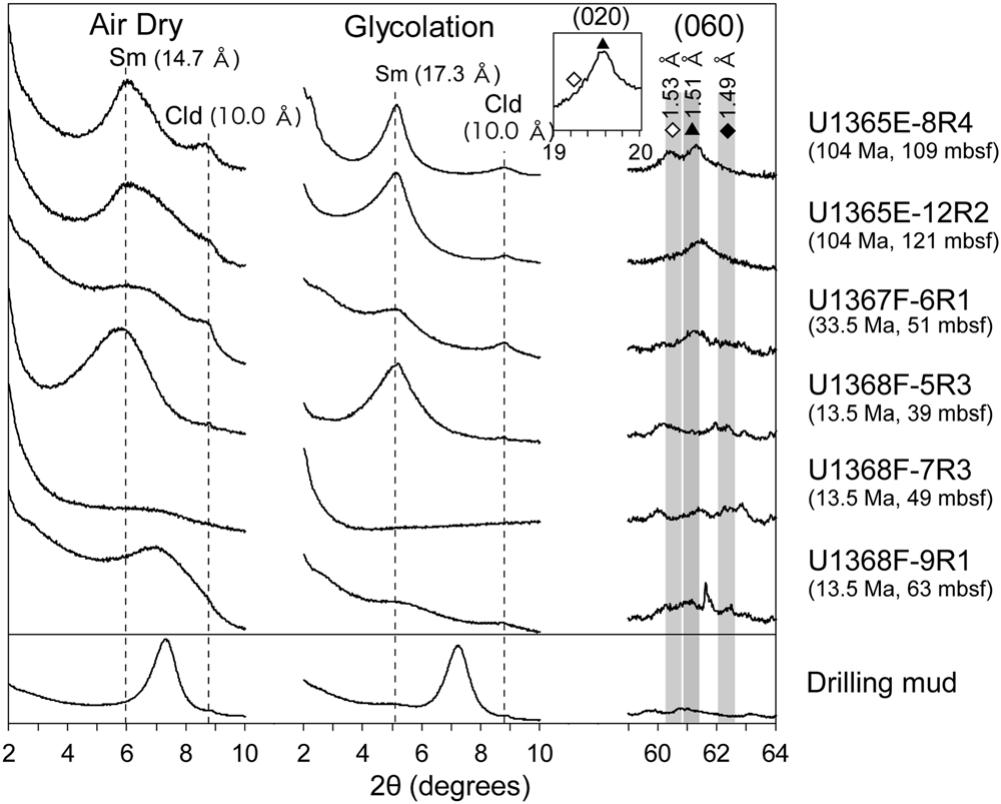
X-ray diffraction (XRD) patterns of clay-sized fractions separated from powdered core samples at Sites U1365, U1367, and U1368 and drilling mud. Low-angle XRD patterns (2*θ*: 2–10°) for air-dried and ethylene-glycolated samples vertically oriented to the *c*-axis of phyllosilicate minerals (left & middle). High-angle XRD patterns (2*θ*: 59–64°) including 060 reflections from randomly oriented samples (right). Symbols: Sm–smectite and Cld–celadonite. In the high-angle XRD patterns, vertical bands show 2*θ* ranges of 060 reflections from trioctahedral phyllosilicate minerals (left with filled diamond), nontronite and celadonite (middle with filled triangle), and dioctahedral phyllosilicate minerals (right with open diamond)^18^. Inserted XRD pattern including a 020 reflection with a filled triangle excludes the assignment of a reflection with a filled diamond to be a 060 reflection from trioctahedral phyllosilicate minerals in U1365E-8R4.

TEM-based clay mineral identification has drawbacks, because basal spacing of smectite minerals is known to shrink under high-vacuum conditions and electron beam bombardment. X-ray diffraction pattern analysis of crystallographically oriented clay minerals with and without intercalation using ethylene glycol is necessary to unambiguously distinguish smectite clays from other non-expandable clays such as celadonite and chlorite^17^. Core samples of U1365E-8R4 and U1365E-12R2 were ground into powder, and clay-sized fractions in the powdered core samples were separated and subjected to TEM-EDS characterisations (Fig. 2c, Supplementary Fig. S2c). In comparison with the clay minerals directly observed in fractures and veins of U1365E-8R4 and U1365E-12R2, the chemical compositions and packet features of the separated clay minerals in the clay-sized fractions were nearly identical (Fig. 2c for U1365-8R4 and Supplementary Fig. S2c for U1365-12R2). As basal spacing increased from 1.5 nm to 1.7 nm after intercalation with ethylene glycol, clay minerals found in U1365E-8R4 and U1365E-12R2 were identified as smectite, with 060 reflections comparable to those of nontronite^18^ (Fig. 3). Given that typical iron contents of nontronite are >30 wt% Fe_2_O_3_^18^, the smectite minerals found in U1365E-8R4 and U1365E-12R2 were not classified to be nontronite (Supplementary Table S3). The iron-deficient smectite with the 060 reflection identical to nontronite is hereafter referred to as nontronite-like smectite.

Next, we extended mineral characterisations to younger basaltic basement cores at Sites U1367 and U1368 (Supplementary Table S2). As shown in Fig. 3, nontronite-like smectite was not detected in three samples from Site U1368 (sample codes: U1368F-5R3, U1368F-7R3, and U1368F-9R1 at depths of 39, 49, and 63 mbsf). Because of low core recovery during drilling (Supplementary Table S1), only one sample was examined for Site U1367 (sample code: U1367F-6R1 at a depth of 51 mbsf), from which an XRD pattern identical to that of nontronite was obtained (Fig. 3). To clarify where nontronite-like smectite was formed in U1367F-6R1, thin sections of rock pieces associated with celadonite-bearing veins were characterised by SEM-EDS (Supplementary Fig. S3). It was revealed that nontronite-like smectite was present along the celadonite-bearing veins, and that the chemical composition of nontronite-like smectite was similar to those found in U1365E-8R4 and U1365E-12R2 (Supplementary Table S3).

## Discussions

In sharp contrast to secondary minerals easily recognised by petrologic observations such as celadonite, saponite, and calcium carbonate, nontronite is technically challenging for mineral identification and localisation in fractures/veins because nontronite is fine-grained and may be interstratified with mica minerals, as has often been speculated^19,20^. Nontronite formation in basaltic basement has been reported from Site 1001 on the Hess Escarpment in the Caribbean Sea (40 km from Deep Sea Drilling Project (DSDP) Site 152), with a crustal age of >76 Ma^19^, and DSDP Site 417 on the Bermuda Rise in the southwestern Atlantic, with a crustal age of 109 Ma^20^. In these previous studies, smectite identification has been performed by XRD analysis of clay-sized fractions separated from powdered core samples. For thin sections of core samples including fractures/veins, SEM-EDS analysis and electron probe microanalysis (EPMA) have been conducted. To our knowledge, no high-resolution TEM studies have compared nanoscale features of clay minerals separated from powdered core samples (typically analysed by XRD) to those prepared from intact core samples as thin sections (typically analysed by SEM-EDS and/or EPMA) to unambiguously identify smectite clays in oceanic crust.

The chemical reactivity and permeability of oceanic crust are generally considered to decline markedly in 10–20 Ma basaltic basement, based on the fact that the increase in the ratio of Fe(III) to total Fe plateaus after >10–20 million years from the time of crust formation^8^. In addition, the ages of fractures/veins filled with calcium carbonate are nearly the same as 10–20 million years from crust formation^21^, which suggests that calcium carbonate is a final alteration product sealing the fractures/veins. Nontronite formation is often observed at deep-sea hydrothermal vents where Fe(II)-bearing hydrothermal fluid reacts with oxygenated seawater at temperatures below 30 °C^22^. Thermodynamic calculations predict that nontronite formation is favourable under oxidising conditions^23^, which agrees with its high Fe(III) content. In basaltic basement, the Si and Fe used in the formation of nontronite-like smectite are derived from the dissolution of basalt during weathering, whereas Na, Ca, K, and Mg are abundantly supplied from circulating seawater. Thus, nontronite-like smectite formation appears to be controlled by the extent to which Si and Fe dissolved from basalt are diluted by circulating seawater in basaltic basement.

To further evaluate the physicochemical differences explaining the presence and absence of nontronite-like smectite in fractures/veins in basaltic basement at Sites U1367 and U1368, it is necessary to consider the chemical composition of the basement fluid and its flow velocity. Although neither of these fluid characteristics was directly measured, the former was extrapolated from the chemical composition of porewater from sediments located immediately above the basaltic basement, given that the basement fluid is recharged from the overlying sediments^15,16,24^ (Supplementary Fig. S4). The latter is generally interpreted based on heat flow data obtained from the seafloor at a drilling site^12^. In principle, heat flow decreases significantly when the heat source in basaltic basement is cooled by vigorous circulation of cold seawater (Supplementary Fig. S5a). In the case of basaltic basement without vigorous seawater circulation, heat flow data from overlying sediments show a well-established cooling model of oceanic crust by conduction rather than convection^25,26^. At Site U1368, heat flow is consistent with the effect of convection-driven cooling (Supplementary Fig. S5a), whereas deviation from the conduction-based model was not evident at Site U1367^12^ (Supplementary Fig. S5b). Thus, it is likely that nontronite-like smectite formation was inhibited by the dilution of basement fluid by vigorous seawater circulation at U1368. If the decreased circulation of seawater in basaltic basement is weak enough to turn the environment anoxic through O_2_ reduction by Fe(II), nontronite-like smectite formation appears to be inhibited^27^. Thus, nontronite-like smectite is present at Site U1367, probably because seawater circulation was not vigorous enough to dilute Fe and Si but was moderate enough to maintain the supply of dissolved O_2_.

**Figure 4:**
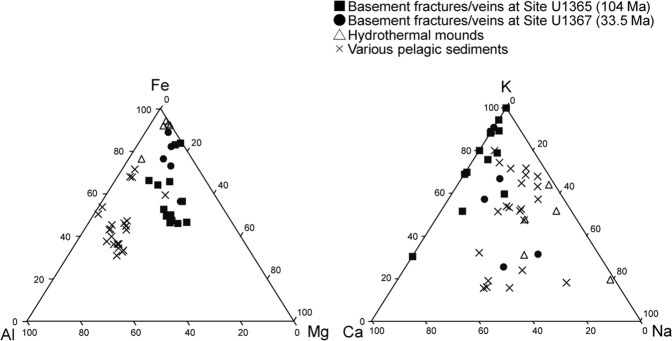
Ternary diagrams of Fe-Al-Mg in sheet structure and K-Ca-Na in interlayer space. Percentages of Fe, Al, and Mg occupying tetrahedral and octahedral layers and those of intercalated K, Ca, and Na are shown on each axis. Plotted data were from hydrothermal mounds located at the Red Seamount near the East Pacific Rise^28^ and from pelagic sediments in the north-central Pacific, Angola Basin, north Fiji Basin, Lau Basin^29^, and from Sites U1365 and U1369^30^.

In comparison with nontronite formed at hydrothermal mounds and found in pelagic sediments^28–30^, the Mg content of nontronite-like smectite at basement fractures/veins was high (Fig. 4). The Fe(III) content of nontronite is high at hydrothermal mounds, whereas high content of Al is characteristic of pelagic sediments. High K content is another characteristic feature of nontronite-like smectite formed in basement fractures/veins. Although high K content might result from interstratification with celadonite, the lack of a Raman spectrum attributed to celadonite and the compositionally homogenous packets in the X-ray elemental mapping images support the inference that K was mainly detected from nontronite-like smectite. The interlayer K likely originated from seawater, because the K content of basaltic lava is generally low^31^. Given that seawater circulates from the overlying sediment into basaltic basement^12^ (Supplementary Fig. S2), the Na/K ratios of ~40 and Ca/K ratios of ~1 at Sites U1367 and U1368 suggest that nontronite-like smectite in the basement fractures/veins selectively takes up K from the surrounding fluid. The preferential fixation of K is explained by the large cell dimension of nontronite that is compatible with the larger ionic radius of K than those of Na and Ca^18^. A previous study argued that high K content is correlated with high Fe(II) content in nontronite formed around the sediment**–**basement interface in oceanic crust^32^. The high content of Fe(II) in the octahedral layer of nontronite typically containing Fe(III) increases the net negative charge of the sheet layer, which seems to be balanced by the increase in the interlayer uptake of K. As the ionic radii are relatively similar between Mg and Fe(II)^33^, it is likely that Mg incorporation into the octahedral layer is attributed to the high K content of nontronite-like smectite in basement fractures/veins.

Nontronite is known to form at hydrothermal mounds, where Fe(III) is abundantly produced from the oxidation of Fe(II) in hydrothermal fluid by O_2_ in seawater^28^ (Supplementary Fig. 6a). The lack of Mg may be caused by mixing of hydrothermal fluid generally depleted in Mg and seawater containing ~50 mmol/kg of Mg^34^ and/or by the incorporation of abundantly produced Fe(III) into the octahedral layer of nontronite. This formation process is typically associated with the emplacement of magma and the vigorous circulation of oxygenated seawater at mid-ocean ridges^10^. Although nontronite may form in oxic zones of subseafloor basaltic lavas at mid-ocean ridges, the circulation of moderately high-temperature fluid enriched in reduced compounds such as HS^−^ and Fe(II)^6^ is likely to transform nontronite into pyrite and saponite on the young ridge flank. If fractures/veins filled with nontronite are impermeable, nontronite may persist against the circulation of the reduced fluid. In this case, the chemical composition of nontronite should be enriched with Fe(III). On the older ridge flank circulated with cold, oxygenated seawater, nontronite-like smectite formation appears to be favourable^7^. However, our results are consistent with the explanation that the intensity of fluid circulation is too high to exceed the concentrations of Si and Fe saturated with nontronite-like smectite. Finally, Mg- and K-enriched nontronite-like smectite forms in basaltic basement without convection-driven fluid circulation. In basement fractures/veins, Mg is likely supplied from circulating seawater and basalt weathering (Supplementary Fig. 6b). Taken all together, we explain that the formation of Mg- and K-enriched nontronite-like smectite in basaltic basement results from basalt weathering rather than hydrothermal alteration, which strongly suggests the possibility that unsedimented basaltic basement is permeable and chemically reactive after 10–20 Ma.

Our results have great implications regarding ocean budgets and an influence on the deep crustal biosphere, given that the basaltic basement overlain by the entirely oxic subseafloor sediment covers 9% to 37% of the total ocean area^13^. In this study, the formation of nontronite-like smectite from crustal fluid indirectly indicates the availability of Fe(II) and O_2_ as the electron donor and acceptor to chemolithotrophic microorganisms. In addition to the cycling of carbon, oxygen and iron, the uptake of K by nontronite-like smectite in unsedimented basaltic basement might affect the global budget of K as an underestimated sink^1^. The upper crust of Mars is composed of old basaltic lava where the presence of liquid water has been reported in the deep subsurface^35^. In addition, subsurface nontronite formation appears to have been widespread on early Mars^36^. With precautions for apparent physicochemical differences such as redox conditions, it is expected that old basaltic lava may be permeable and reactive to host extant life and/or signatures from past life in fractures/veins filled with iron-rich smectite on Mars.

## Conclusion

In this study, we investigated basaltic basement thinly covered with sediments to clarify the permeability and reactivity of aged oceanic crust. At the rims of fractures/veins filled with common secondary minerals such as celadonite and iron oxyhydroxides, nontronite-like smectite was found to form in 33.5- and 104-Ma basaltic basement, which is likely explained by basalt-seawater interactions without vigorous fluid circulation. As it is technically feasible to combine high-resolution TEM characterisations of powdered and intact core samples to clarify the formation process of smectite, further efforts are necessary to expand the spatial and temporal coverage and estimate the global impact of basalt weathering in aged oceanic crust.

## Methods

### Sample collection

Rock core samples were collected from basaltic basement at Sites U1365, U1367, and U1368 in the SPG during IODP Expedition 329 (October 9 through December 13, 2010) (Supplementary Fig. S1). The formation ages of basaltic basement at the three sites range from 13.5 Ma to 104 Ma, and the thicknesses of sediment cover range from 16 to 75 m (Supplementary Table S1).

### Thin section preparation and light microscopy

To clarify mineral composition within rock fractures, thin sections were prepared. Fracture/vein-bearing core samples were dehydrated twice in 100% ethanol for 5 min, and core samples were infiltrated four times with LR White resin (London Resin Co. Ltd., Aldermaston, England) for 30 min and solidified in an oven at 50 °C for 48 h. Solidified blocks were trimmed into thin sections and polished with corundum powder and diamond paste. Mineral assemblages and textures were observed using an optical microscope (BX51; Olympus) with a charge-coupled device (CCD) camera (DP71; Olympus).

### Scanning electron microscopy (SEM) and µ-Raman spectroscopy

Back-scattering electron images of thin sections coated with carbon were obtained by field emission scanning electron microscopy (FE-SEM) using a Hitachi FE-SEM S-4500 instrument (Tokyo, Japan) at an accelerating voltage of 15 kV and an emission current of 15 µA. Energy-dispersive X-ray spectroscopy (EDS) was used to analyse chemical compositions of mineral phases according to image contrasts. Micro-Raman spectroscopy with Ion Laser Technology 5500 A (Tokyo, Japan) was used to characterised the fracture/vein-infilling minerals. Raman spectra from fracture/vein-filling minerals were obtained using a 50-cm single polychromator imaging spectrometer (Bruker Optics, Osaka, Japan), which was equipped with an optical microscope (BX51; Olympus), an Ar^+^ laser (514.5 nm, 5,500 A; International Light Technologies, Peabody, MA, USA), and a CCD camera (1024 × 256 pixels; DU401A-BR-DD; Andor Technology, Belfast, Ireland). The incident laser was operated at 20 mW, and the spatial resolution was ~1 μm. An edge-cut filter was used to remove the Rayleigh line. Raman lines of naphthalene at 513.6 cm^−1^, 763.5 cm^−1^, 1,021.3 cm^−1^, 1,147.3 cm^−1^, 1,382.3 cm^−1^, 1,464.3 cm^−1^, and 1,576.3 cm^−1^ were used to calibrate Raman shift. Spectral resolution was ~1 cm^−1^. Raman spectra were interpreted based on comparison with those obtained from RRUFF^37^ (http://rruff.info).

### Transmission electron microscopy (TEM) and scanning transmission electron microscopy (STEM)

Focused ion beam (FIB) milling sample preparation technology (Hitachi FB-2100 instrument, Tokyo, Japan) was used to excise a region of interest at the rims of fractures/veins in rock cores. Transmission electron microscopy (TEM) was used to examine the structure and composition of minerals at the nanometre scale. JEOL JEM-2010 (Tokyo, Japan) with energy dispersive X-ray spectrometry (EDS) was operated at 200 kV. Elemental compositions of phyllosilicate minerals were quantified with K-factors of Al/Si, Ti/Si, Fe/Si, Mn/Si, Mg/Si, Ca/Si, Na/Si, and K/Si, which were calculated from EDS spectra based on JCSS-3110 montmorillonite^38^. To clarify elemental distributions at the submicron scale, the FIB sections were examined by high-angle annular dark field scanning transmission electron microscopy (HAADF-STEM) and STEM-EDS X-ray elemental mapping. JEOL JEM-ARM200F STEM was operated at an accelerating voltage of 200 kV at the Kochi Institute for Core Sample Research of the Japan Agency for Marine-Earth Science and Technology (JAMSTEC).

### X-ray diffraction (XRD) pattern analysis

The portions of rock cores examined as described above were powdered using a mortar and pestle. Clay-sized fractions in powdered samples were suspended in distilled and deionised water, centrifuged at 3,000 rpm for 5 min, and then freeze-dried for storage. To identify phyllosilicate minerals, X-ray diffraction (XRD) pattern analysis was performed. RIGAKU RINT-ULTIMA-2100 (Tokyo, Japan) was operated at 40 kV and 30 mA with monochromatised Cu-Kα radiation. Freeze-dried samples were suspended in distilled and deionised water and mounted on glass slides to orient the sheet structure parallel to the glass slide. Air-dried and ethylene-glycolated samples were scanned by X-ray in a 2*θ* range of 2–10°. Randomly oriented samples were examined to determine the 060 reflection and clarify the sheet structure.

### TEM characterisation of oriented phyllosilicate samples

Clay-sized fractions examined by XRD analysis were crystallographically oriented and embedded in LR White resin. For TEM-EDS, ultra-thin sections perpendicularly containing the sheet structure were prepared with an ultramicrotome (Ultracut S, Reichert-Nissei, Tokyo, Japan).

## Supplementary information

Supplementary Information
