# Supplementary material for: Iron-rich Smectite Formation in Subseafloor Basaltic Lava in Aged Oceanic Crust
Source: Sci Rep. 2019 Aug 5;9:11306. doi: 10.1038/s41598-019-47887-x (PMC6683296; doi:10.1038/s41598-019-47887-x)
Supplement: Supplementary file 1 — Supplementary Information [file 41598_2019_47887_MOESM1_ESM.docx]

**Supplementary Information**

**Iron-rich Smectite Formation in Subseafloor Basaltic Lava in Aged Oceanic Crust**

Seiya Yamashita^1^, Hiroki Mukai^1^, Naotaka Tomioka^2^, Hiroyuki Kagi^3^, Yohey Suzuki^1*^

^1^Department of Earth and Planetary Science, The University of Tokyo, 7-3-1 Hongo, Bunkyo-ku, Tokyo, Japan

^2^Kochi Institute for Core Sample Research, Japan Agency for Marine-Earth Science and Technology (JAMSTEC), 200 Monobe Otsu, Nankoku, Kochi 783-8502, Japan

^3^Department of Chemistry, The University of Tokyo, 7-3-1 Hongo, Bunkyo, Tokyo 113-0033, Japan

Supplementary Figure S1–S8

Supplementary Table S1–S3


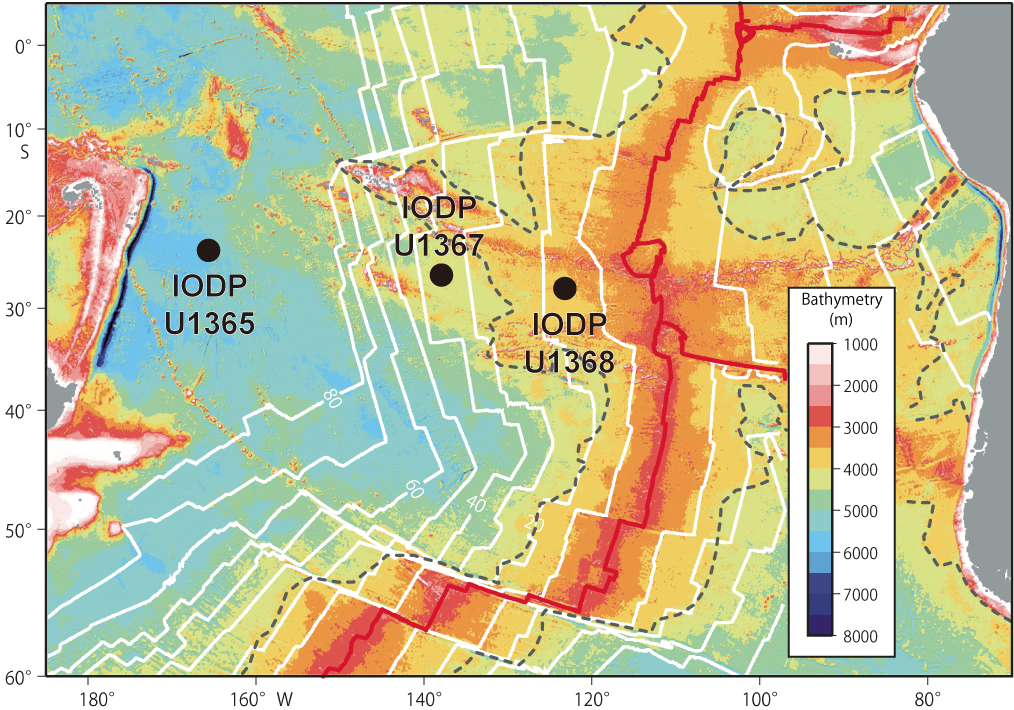
**Supplementary Figure S1.** A bathymetry map showing drilling locations modified after the reference 12.

**
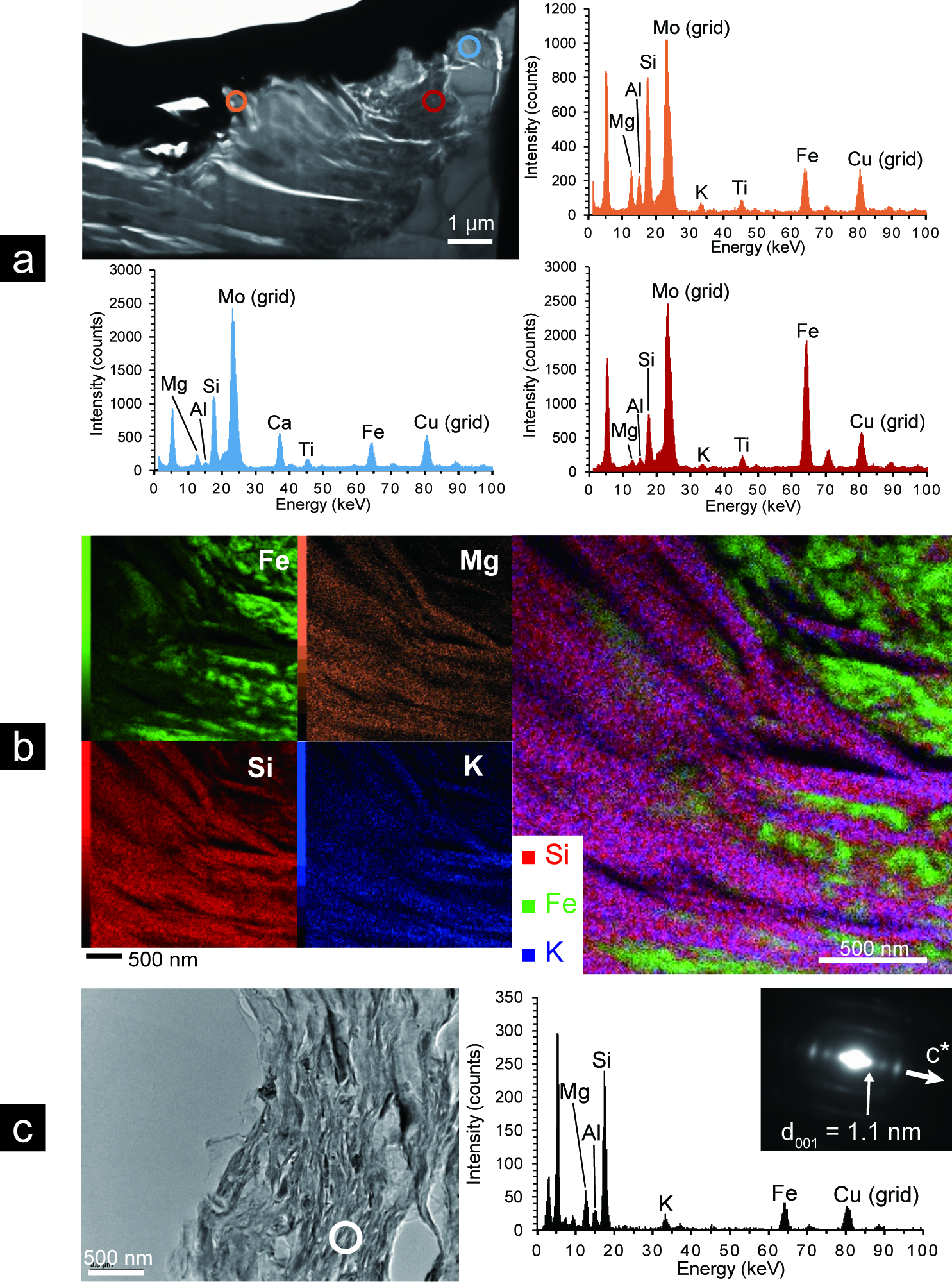
Supplementary Figure S2. Mineralogical characteristics of Fe-rich phyllosilicate minerals in U1365E-12R2.** (**a**) Bright-field TEM micrograph of fibrous material located between an iron-oxyhydroxide-filled vein and the groundmass. EDS spectra were obtained from circles with the same color. (**b**) Elemental mapping images of Fe, Si, Mg, and K by scanning transmission electron microscopy and an RGB synthesis image of Si, Fe and K. (**c**) Bright-field TEM micrograph of a suspended fraction of a powdered sample with a white circle, from which an SAED pattern and an EDS spectrum were obtained.

**
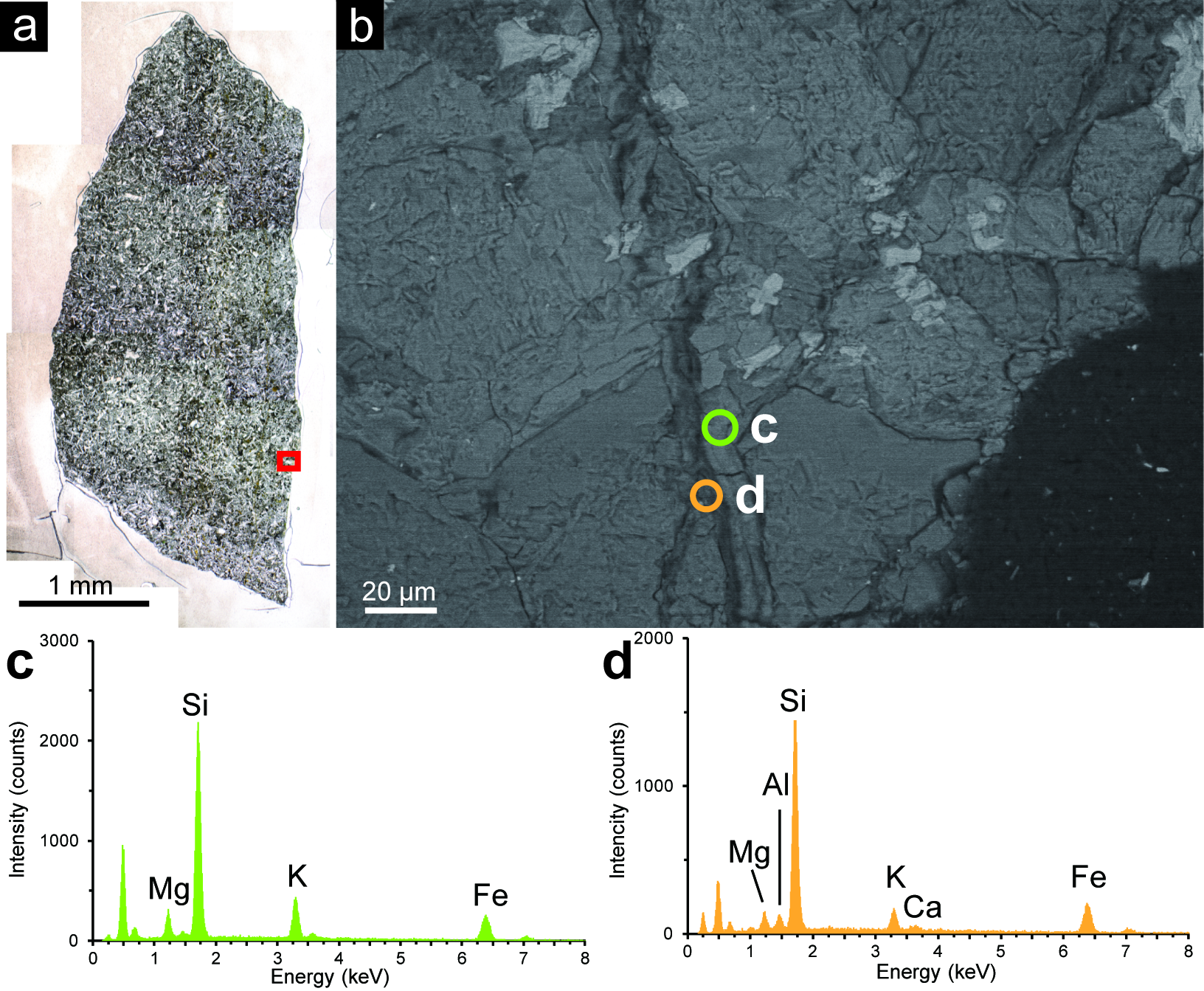
Supplementary Figure S3.** **Mineralogical characteristics of phyllosilicate minerals in U1367F-6R1.** (**a**) Optical microscopy image of a thin section of a rock piece with veins. (**b**) Back-scattered electron image of mineral-filled veins with green (**c**) and orange (**d**) circlers from which EDS spectra were obtained.

**
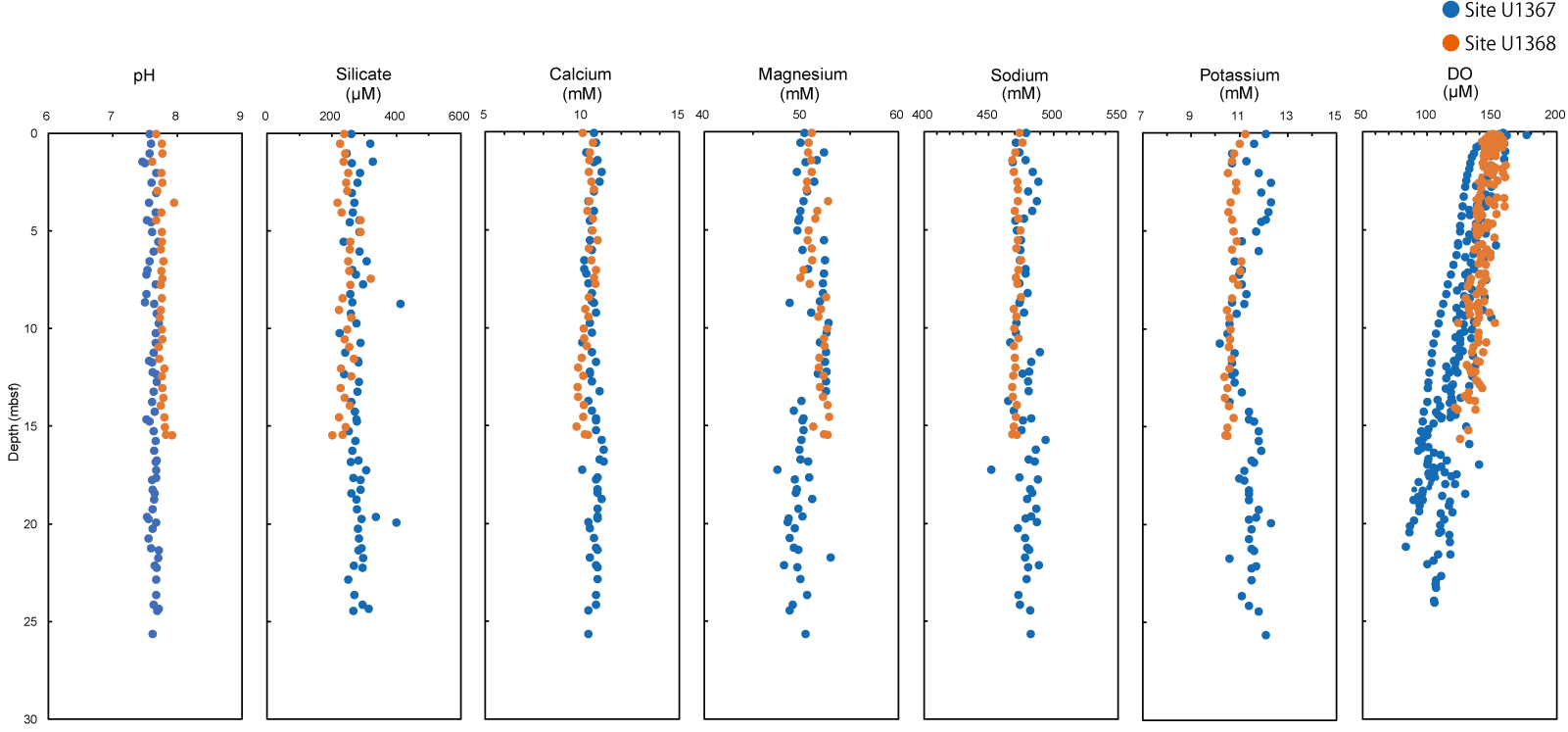
Supplementary Figure S4.** Depth profiles of porewater chemistry in sediments at Sites U1367 and U1368 showing pH and concentrations of silicate, calcium, magnesium, sodium, potassium, and dissolved oxygen (DO) ^15,16^.


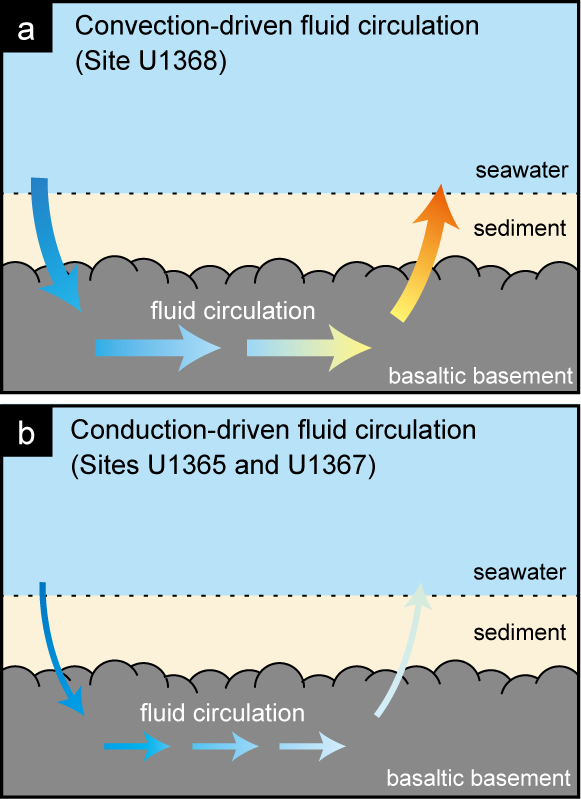
**Supplementary Figure S5.** Schematic illustrations of convection- (**a**) versus conduction-driven fluid flows (**b**) in aged oceanic crust, which was inferred from heat flow data from the seafloor at Sites U1365, U1367, and U1368^12^. The extent of fluid flow is expressed as arrows: large, bold arrows showing strong fluid flow and small, thin arrows showing weak fluid flow.


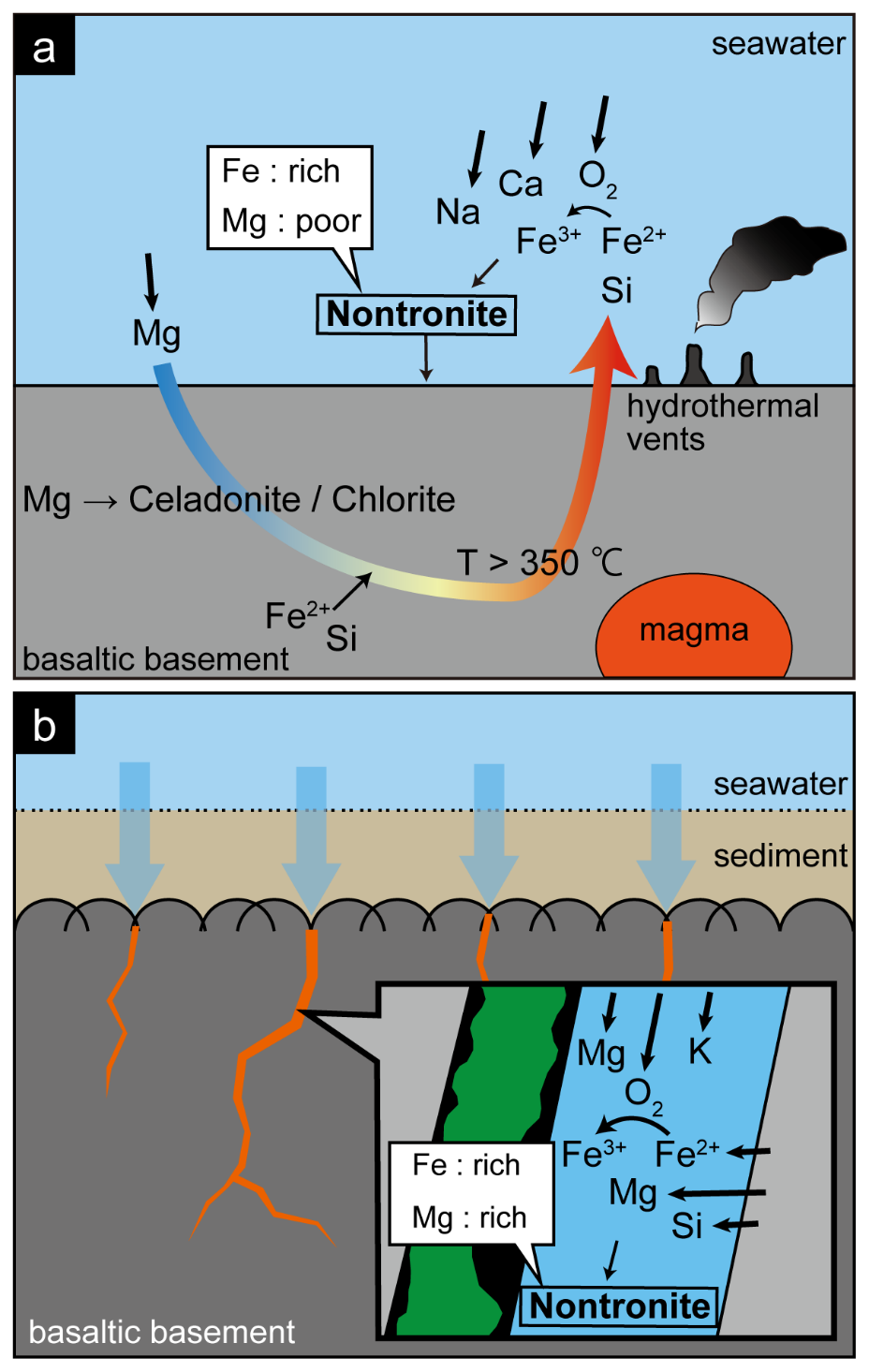
**Supplementary Figure S6.** Schematic illustrations of formation processes of nontronite by hydrothermal alteration near the seafloor (**a**) versus by weathering in subsurface basaltic lava (**b**).

**Supplementary Table S1.** Crustal ages, sediment thicknesses and basement core recoveries at Sites U1365^24^, U1367^15^, and U1368^16^.

|  | Site U1365 | Site U1367 | Site U1368 |
| --- | --- | --- | --- |
| Crustal age | 104 Ma | ~33.5 Ma | ~13.5 Ma |
| Sediment thickness | 75 m | 26 m | 16 m |
| Basement core recovery | 74.6 % | 11 % | 27.6 % |

**Supplementary Table S2.** Photographic images and visual descriptions of basaltic core samples on shipboard at Sites U1365, U1367, and U1368.

| U1365E-8R4 | Description |
| --- | --- |
| 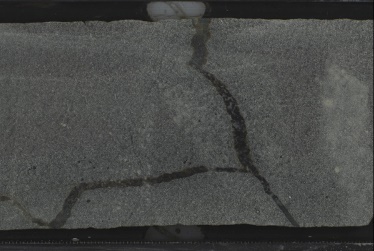 | Aphyric microcrystalline to fine grained massive basalt with a gray to green background (very slight celadonite alteration). One celadonite vein with minor pyrite is present through the center of the rock, which is flanked by a narrow and diffuse dark green halo (celadonite). Piece length is 10 cm. |
| U1365E-12R2 | Description |
| 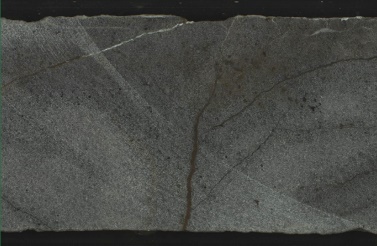 | Aphyric microcrystalline massive basalt with dark gray and brown background alteration. Mixed brown and dark green alteration halos flank one 0.1 to 0.3 mm branching iron-oxyhydroxide (vertical) and celadonitic (horizontal) veins. Alteration patches of celadonite and iron-oxyhydroxide are present. Piece length is 8 cm |
| U1367F-6R1 | Description |
| 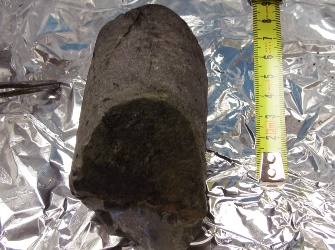 | Aphyric microcrystalline to cryptocrystalline basalt with green to gray background alteration that varies from slight to moderate. Veins are steeply dipping including a mixed celadonite (20% of vein fill), iron-oxyhydroxide at (80% of vein fill) vein (0.3mm wide) that is flanked by a brown (iron-oxyhydroxide) and dark green (celadonite) halo, and two celadonite veins (0.2mm wide). |
| U1368F-5R3 | Description |
| 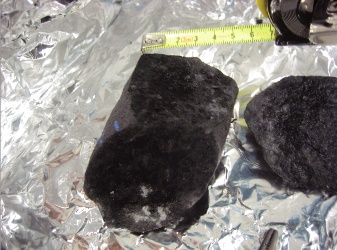 | Sparsely phyric microcrystalline massive basalt with slight dark green, pervasive alteration. Dark green celadonitic vein is present. Basaltic groundmass is sparsely vesicular with celadonite filling vesicles. |
| U1368F-7R3 | Description |
| 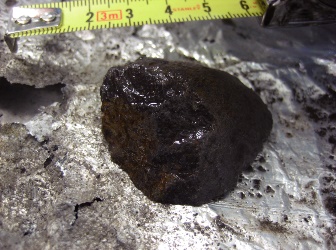 | Slightly altered massive microcrystalline to cryptocrystalline sparsely phyric basalt. Alteration is pervasive and it consists of iron-oxyhydroxides and celadonite within the whole rock. The vein is composed of iron-oxyhydroxides (curved and 0.1 mm thick with a narrow brown/red halo. Vesicles are filled with celadonite and iron-oxyhydroxides. |
| U1368F-9R1 | Description |
| 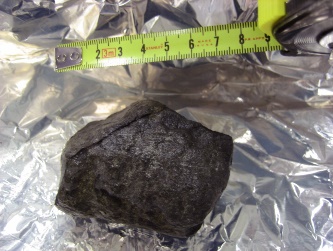 | Aphyric microcrystalline basalt with brown/green gray background that represents slight to moderate alteration. Alteration consists of celadonite and iron-oxyhydroxides. Vein is vertical and composed of iron-oxyhydroxides (90% and clay minerals 10%). Vein thickness is 0.4mm and it is not flanked by a halo. Vesicles are filled with celadonite and iron-oxyhydroxides and there are ~2-4% of vesicles in the whole rock. |

| Site | U1365 | U1365 | U1365 | U1365 |  | U1367 | U1367 | U1367 | U1367 | U1367 |
| --- | --- | --- | --- | --- | --- | --- | --- | --- | --- | --- |
| Core Unit | 12R2 | 12R2 | 12R2 | 12R2 |  | 6R1 | 6R1 | 6R1 | 6R1 | 6R1 |
| SiO_2_ | 41.17 | 54.36 | 55.08 | 50.99 |  | 41.01 | 59.96 | 55.44 | 55.94 | 53.50 |
| Al_2_O_3_ | 12.15 | 8.42 | 6.19 | 8.64 |  | 2.35 | 5.00 | 3.26 | 3.97 | 1.08 |
| TiO_2_ | 1.95 | 0.35 | 0.20 | 0.10 |  | 2.31 | 0.47 | 0.94 | 0.58 | 0.64 |
| Fe_2_O_3_ | 25.97 | 19.98 | 17.06 | 26.89 |  | 38.57 | 19.69 | 24.58 | 28.19 | 34.31 |
| MnO | 0.00 | 0.00 | 0.00 | 0.00 |  | 1.23 | 0.15 | 0.00 | 0.00 | 0.00 |
| MgO | 14.36 | 11.48 | 13.39 | 5.12 |  | 6.00 | 10.21 | 5.82 | 4.29 | 3.25 |
| CaO | 0.62 | 2.19 | 1.69 | 1.57 |  | 3.27 | 1.02 | 2.93 | 1.33 | 0.66 |
| K_2_O | 3.47 | 2.80 | 4.82 | 6.26 |  | 2.17 | 1.42 | 5.69 | 4.71 | 6.56 |
| Na_2_O | 0.31 | 0.42 | 1.57 | 0.43 |  | 3.09 | 2.07 | 1.33 | 1.00 | 0.00 |
| sum | 100.00 | 100.00 | 100.00 | 100.00 |  | 100.00 | 100.00 | 100.00 | 100.00 | 100.00 |

**Supplementary Table S3.** Compositions of nontronite in fractures/veins of basaltic basements at Sites U1365 and U1367 quantified by TEM-EDS and SEM-EDS, respectively.

| Site | U1365 | U1365 | U1365 | U1365 | U1365 | U1365 |  | U1365 | U1365 | U1365 |
| --- | --- | --- | --- | --- | --- | --- | --- | --- | --- | --- |
| Core Unit | 8R4 | 8R4 | 8R4 | 8R4 | 8R4 | 8R4 |  | 12R2 | 12R2 | 12R2 |
| SiO_2_ | 62.74 | 52.82 | 52.65 | 49.94 | 57.10 | 59.42 |  | 43.25 | 41.37 | 43.80 |
| Al_2_O_3_ | 4.81 | 5.78 | 9.20 | 8.10 | 1.15 | 0.27 |  | 11.66 | 12.31 | 11.59 |
| TiO_2_ | 0.04 | 0.09 | 0.40 | 0.38 | 0.48 | 0.49 |  | 0.84 | 1.20 | 0.85 |
| Fe_2_O_3_ | 22.90 | 23.83 | 20.55 | 27.45 | 31.82 | 28.85 |  | 27.42 | 24.50 | 24.87 |
| MnO | 0.00 | 0.00 | 0.00 | 0.00 | 0.00 | 0.00 |  | 0.00 | 0.00 | 0.00 |
| MgO | 7.15 | 12.63 | 14.93 | 7.23 | 5.33 | 5.34 |  | 12.91 | 16.05 | 16.02 |
| CaO | 0.28 | 0.54 | 1.57 | 2.06 | 0.33 | 0.32 |  | 0.78 | 1.42 | 0.00 |
| K_2_O | 2.09 | 4.30 | 0.69 | 4.83 | 3.68 | 5.31 |  | 3.13 | 3.15 | 2.87 |
| Na_2_O | 0.00 | 0.00 | 0.00 | 0.02 | 0.11 | 0.00 |  | 0.00 | 0.00 | 0.00 |
| sum | 100.00 | 100.00 | 100.00 | 100.00 | 100.00 | 100.00 |  | 100.00 | 100.00 | 100.00 |
